# Relationship between non-suicidal self-injury and sleep among college students: a multifactorial analysis

**DOI:** 10.3389/fpsyt.2025.1584008

**Published:** 2025-06-13

**Authors:** Yiting Zhao, Yue Sun

**Affiliations:** ^1^ Children’s Hospital, Tianjin University, Tianjin, China; ^2^ Tianjin Union Medical Center, Tianjin, China

**Keywords:** non-suicidal self-injury, sleep disturbances, college students, hypnotic medications, psychosocial factors

## Abstract

**Objective:**

To investigate the relationship between non-suicidal self-injury (NSSI) and sleep quality, and associated psychosocial factors among college students.

**Methods:**

A cross-sectional study was conducted using cluster sampling at a university in Tianjin, China, in August 2022. Validated questionnaires, including the Adolescents Self-Harm Scale (ASHS), Pittsburgh Sleep Quality Index (PSQI), and Center for Epidemiological Studies Depression Scale (CES-D), were administered to 454 freshmen. Generalized linear models were employed to analyze associations between NSSI and independent variables.

**Results:**

After excluding the questionnaires with insincere responses based on the test time and trap questions, a total of 454 valid questionnaires were collected. The use of hypnotic medications was significantly associated with higher NSSI scores. (β=2.342,P<0.001). Sleep duration was positively associated with NSSI scores (β=0.765,P=0.017). CES-D score positively affected NSSI score (β=0.117,P<0.001). Non-heterosexual students scored 2.379 points higher on the NSSI scale than heterosexual students (P<0.001). Relationship with parents significantly positively affects NSSI. The score of NSSI was significantly increased by 2.951 (P=0.002) in those who had a poor father-child relationship compared to those with harmonious parental relationships. Childhood trauma had a significant positive effect on NSSI, that is, students who had experienced childhood trauma had 3.815 higher NSSI scores than students who had not experienced trauma (P<0.001).

**Conclusion:**

Non-suicidal self-injury (NSSI) among college students is associated with sleep disturbances, particularly reduced sleep duration and the use of hypnotic medications. Moreover, psychosocial risk factors, including depression, non-heterosexual identity, familial discord, and childhood trauma, are also linked to the occurrence of NSSI. Intervention measures that improve sleep quality and provide psychosocial support may effectively reduce the risk of non-suicidal self-injury behavior.

Non-suicidal self-injury (NSSI) is defined as the intentional and repetitive act of inflicting harm on one’s own body without the intent to cause death (1). Common manifestations of NSSI include cutting or stabbing the skin, pulling out hair, hindering wound healing, self-administering medication, and physically striking oneself (2, 3). In recent years, the incidence of NSSI has shown a significant upward trend globally (4, 5). And this phenomenon is also quite prevalent among college students (6, 7). Meanwhile, sleep disorders persist as a major concern among college students (8). Previous research has consistently demonstrated a strong association between NSSI and sleep conditions (9). A too short sleep duration can elevate the risk of NSSI (10, 11). Both insomnia (12) and poor sleep (13) quality can significantly increase the likelihood of NSSI. However, research exploring the relationship between sleep and NSSI remains scarce, leaving the specific associations between sleep parameters and NSSI poorly understood. We designed this study to examine the prevalence of NSSI among college students. Also ,we aim to explore the bidirectional impact between NSSI and sleep conditions, and figure out which risk factors are closely linked to NSSI.

## 1 Objects and methods

### 1.1 Objects

The subjects of the study were students enrolled in a university in Tianjin at the end of August 2022. In the context of psychological counseling lectures for freshmen, counselors distributed questionnaires. Utilizing cluster sampling, a total of 500 questionnaires were administered. After excluding insincere responses (determined by test duration and trap questions), 454 valid questionnaires were retained, and the effective recovery rate reaches 90.8%. For this survey, after obtaining consent from the faculty teachers, the purpose and usage of the questionnaire were explained in advance to the counselors. During the investigation, explain the purpose of the investigation to the students and inform them that they can stop filling in at any time.

### 1.2 Tools

#### 1.2.1 Demographics and psychosocial factors

After consulting relevant literature, self-compiled questions were used to understand the general situation of students, including gender, age, whether they are local, the education level of both parents, the main living person, the relationship with parents, personality preference, sexual orientation, whether they have had traumatic experiences such as childhood abuse and neglect, and birth order.

#### 1.2.2 The adolescents self-harm scale

ASHS questionnaire was employed in this study, having been revised by domestic scholars who evaluated its reliability and validity. This revised questionnaire is more appropriate for Chinese adolescents and children. In this survey, the Cronbach’s α coefficient for the questionnaire was calculated to be 0.926. Additionally, the Bartlett’s test of sphericity produced a p-value of less than 0.05, indicating significant results. The Kaiser-Meyer-Olkin (KMO) measure of sampling adequacy was found to be 0.748.

#### 1.2.3 The Pittsburgh sleep quality index

PSQI was utilized to evaluate sleep quality. In this survey, the overall Cronbach’s α coefficient for the scale, excluding questions 1 and 3, was found to be 0.797. Additionally, the Bartlett’s test of sphericity yielded a significance level of P < 0.05, and the Kaiser-Meyer-Olkin (KMO) value was determined to be 0.784.

#### 1.2.4 The center for epidemiological survey depression scale

CES-D was employed to screen for depression. The overall Cronbach’s α reliability coefficient for the scale was 0.812, while Bartlett’s test of sphericity indicated a significance level of P < 0.05, and the KMO value was recorded at 0.929.

### 1.3 Methods

Data were imported into SPSS26.0 from the Questionnaire Star platform. The samples were described using frequencies or percentages. Categorical data were compared using the chi-square test, while continuous data were analyzed using either parametric or non-parametric tests, depending on the results of normality tests. Regression models were selected based on the types of data to explore the influencing factors. In this study, all P-tests were conducted as two-tailed tests, and a P value of less than 0.05 was deemed statistically significant. All items on the scales were subjected to Harman’s single-factor test. The results indicated that there were 17 factors with eigenvalues greater than 1, with the maximum factor variance explained being 21.3%, which is below the 40% threshold. Consequently, no significant common method bias was detected.

## 2 Results

### 2.1 Basic information

The participants in this study are freshmen from the Class of 2022, with an average age of 18.5 years. The gender distribution is 54.6% male and 45.4% female. The ASHS questionnaire identified 65 individuals, representing 14.3%, as having NSSI. The CES-D scale indicated that 96 individuals scored above 15, which corresponds to 21.1% of the sample. Furthermore, the PSQI scale revealed that 73 individuals achieved a total PSQI score greater than 7, constituting 16.1% of the participants (see **Tables 1**, **2**).

**Table 1:** Basic information distribution.

Variables	Groups	n (%)
Gender	Male	248 (54.6)
	Female	206 (45.4)
Sexual orientation	Heterosexual	389 (85.7)
	Non-heterosexual	65 (14.3)
personality trait	Slightly introverted	118 (26.0)
	Slightly neutral	238 (52.4)
	Slightly Extroverted	98 (21.6)
native person	Yes	123 (27.1)
	No	331 (72.9)
Have had traumatic childhood experiences	Yes	52 (11.5)
	No	402 (88.5)
Situation of birth	Without siblings	182 (40.1)
	With siblings (First born)	149 (32.8)
	With siblings (Not the first born)	123 (27.1)
Who the main ones live with	both parents	374 (82.4)
	One of the parents	42 (9.3)
	Other relatives	38 (8.3)
Father's education level	Primary school and below	59 (13.0)
	Junior high school	173 (38.1)
	High school	109 (24.0)
	University and above	113 (24.9)
Mother's education level	Primary school and below	87 (19.2)
	Junior high school	160 (35.2)
	High school	116 (25.6)
	University and above	91 (20.0)
Relationship with parents	Positive relationship with father, strained relationship with mother	14 (3.1)
	Positive relationship with mother, strained relationship with father	29 (6.4)
	Good relations with both parents	401 (88.3)
	Poor relations with both parents	10 (2.2)

**Table 2:** Detection results of Students.

Name of questionnaire	Groups	n (%)
ASHS	Non-NSSI group	389 (85.7)
	NSSI group	65 (14.3)
CES-D	≤15 score	358 (78.9)
	16-19 score	36 (7.9)
	≥20 score	60 (13.2)
PSQI	≤7 score	381 (83.9)
	≥8 score	73 (16.1)

### 2.2 Analysis of factors influencing NSSI

In the univariate analysis, differences in NSSI among college students were observed across various factors, including gender, sexual orientation, personality traits, experiences of childhood trauma, and parental relationships. There was no statistical difference in NSSI between local origin, birth status, main breadwinner and parents’ education level (**Table 3**).

**Table 3:** Analysis of differences between the relevant questions and the NSS.

Variables	Groups	Z/H	P
Gender	Male	-2.712	0.007
	Female		
Situation of birth	Without siblings	2.451	0.294
	With siblings (First born)		
	With siblings (Not the first born)		
Who the main ones live with	both parents	0.963	0.618
	One of the parents		
	Other relatives		
Father's education level	Primary school and below	0.324	0.955
	Junior high school		
	High school		
	University and above		
Mother's education level	Primary school and below	1.833	0.608
	Junior high school		
	High school		
	University and above		
Relationship with parents	Positive relationship with father, strained relationship with mother	15.642	0.001
	Positive relationship with mother, strained relationship with father		
	Good relations with both parents		
	Poor relations with both parents		
native person	Yes	-0.506	0.613
	No		
Have had traumatic childhood experiences	Yes	-6.388	0.000
	No		
personality trait	Slightly introverted	14.755	0.001
	Slightly neutral		
	Slightly Extroverted		
Sexual orientation	Heterosexual	-4.479	0.000
	Non-heterosexual		

ASHS scores were taken as dependent variables and sleep scores as independent variables. Other relevant variables are control variables. Since ASHS scores are continuous but not normally distributed data, the generalized linear model is an extension of the general linear model and does not forcibly change the measurement of the data. A generalized linear model (well-fitted, P< 0.05) was used to analyze associations. After controlling for depression and other factors, NSSI was not significant in the correlation test with a single sleep time factor, but it was significant in the model with the addition of influencing factors, which may have a certain depressing effect. Generalized linear models were selected based on model fit indices and data distribution. There exists a significant correlation between NSSI and sleep duration (β = 0.765, P = 0.017). Additionally, a significant relationship between NSSI and the use of hypnotic drugs persists (β = 2.342, P < 0.05). Among the control variables, the CES-D score exhibited a significant positive influence on the ASHS score (P < 0.05), with a coefficient of 0.117. This indicates that higher scores on the depression scale, which reflect more severe depressive states, correspond to higher ASHS scores. Sexual orientation plays a significant role in NSSI, as evidenced by non-heterosexual students scoring an average of 2.379 points higher on the ASHS scale than their heterosexual counterparts (P < 0.05). The relationship with parents significantly and positively influences NSSI, indicating that individuals with a harmonious relationship with their mother, but not with their father, score 2.951 points higher on the Adolescent Self-Harm Scale (ASHS) compared to those with harmonious relationships with both parents (P=0.002). Additionally, childhood experiences have a significant and positive impact on NSSI, as students who have encountered trauma-related experiences in childhood score 3.815 points higher on the ASHS than those without such experiences (P<0.05) (**Table 4**).

**Table 4:** Generalized linear regression model.

Variables	Groups	B	SE	95%CI	Waldχ^2^	P
(intercept)		-1.49	1.584	-4.60~1.62	0.884	0.347
Sleep quality		-0.297	0.403	-1.09~0.49	0.543	0.461
Sleep latency		-0.098	0.359	-0.8~0.61	0.074	0.785
Sleep duration		0.765	0.321	0.14~1.40	5.679	0.017
Sleep efficiency		-0.671	0.399	-1.45~0.11	2.834	0.092
Sleep disturbances		0.416	0.482	-0.53~1.36	0.744	0.388
Use of sleeping medication		2.342	0.647	1.07~3.61	13.1	0.000
Daytime dysfunction		-0.176	0.287	-0.74~0.39	0.376	0.540
Having nightmares	None	-0.159	1.393	-2.89~2.57	0.013	0.909
	<1time/week	-0.143	1.418	-2.92~2.64	0.01	0.920
	1-2times/week	1.333	1.668	-1.94~4.60	0.639	0.424
	≥3times/week	0				
CES-D score		0.117	0.030	0.06~0.18	14.876	0.000
Gender	Male	-0.396	0.482	-1.34~0.55	0.673	0.412
	Female	0				
Sexual orientation	Non-heterosexual	2.379	0.681	1.05~3.71	12.211	0.000
	Heterosexual	0				
personality trait	Slightly introverted	0.959	0.673	-0.36~2.28	2.029	0.154
	Slightly neutral	0.405	0.579	-0.73~1.54	0.488	0.485
	Slightly Extroverted	0				
Relationship with parents	Positive relationship with father, strained relationship with mother	2.353	1.353	-0.30~5.01	3.025	0.082
	Positive relationship with mother, strained relationship with father	2.951	0.959	1.07~4.83	9.469	0.002
	Poor relations with both parents	-2.464	1.610	-5.62~0.69	2.343	0.126
	Good relations with both parents	0				
Have had traumatic childhood experiences	Yes	3.815	0.779	2.29~5.34	23.984	0.000
	No	0				

## 3 Discussion

In this survey, 14.3% of college students exhibited non-suicidal self-injury behavior, while 16.1% experienced sleep disorders. Both NSSI and sleep disorders are significant issues affecting the college student population.

After controlling for relevant variables, a significant positive influence of sleep duration on NSSI was observed. This study finds that shorter sleep duration is more likely to be associated with the occurrence of NSSI, which aligns with previous research. For example, in a survey of 40 patients diagnosed with borderline personality disorder, the established generalized model indicates that both sleep deprivation and prolonged bed rest may elevate the risk of NSSI (10). A large-scale Norwegian study found that the average sleep duration of youths reporting NSSI was 5.33 hours, which is 0.96 hours shorter than that of those who did not report NSSI (11).

As this is a cross-sectional study, it remains uncertain whether short sleep duration is a direct factor contributing to NSSI. Nevertheless, it is clear that adequate sleep is beneficial for the physical and mental health of college students (14, 15).

Secondly, this study has found that a higher frequency of hypnotic drug use may be associated with an increased risk of NSSI. Currently, there are few studies focusing on the use of hypnotic drugs among college students. However, it is known that irregular and frequent use of hypnotic drugs occurs within this population. However, the long-term irregular use of hypnotic drugs not only exacerbates chronic sleep problems but may also lead to drug dependence (16). Therefore, schools should strengthen the dissemination of sleep health knowledge and guide college students in developing good sleep habits (17). Simultaneously, colleges and universities must pay special attention to students with sleep disorders and provide timely assistance and interventions as needed. Finally, this study found that depressive states, sexual orientation, the relationship with parents, especially the relationship with the father, and traumatic experiences such as abuse and neglect in childhood can all significantly affect self-harm (18). To prevent and reduce the occurrence of NSSI among college students, the support of multiple parties such as schools, families, and society is required. Schools can actively carry out popular science activities and publicize the importance of seeking medical treatment to reduce the stigma associated with medical treatment. They should help students reasonably vent their emotions and stress, conduct regular screenings of psychological states, pay attention to students with risk factors related to NSSI, and promptly guide students to seek medical treatment. Family members, especially fathers, should communicate more with their children, establish good parent-child relationships, and pay attention to the psychological changes of children during the growth process. Society is advised to provide multiple channels to make it convenient for students to seek psychological counseling.

## 4 Limitations

Firstly, due to certain limitations, the sample size of this survey is relatively small. Furthermore, the sample consists exclusively of freshmen from a university in the Tianjin region. This limitation restricts the generalizability of the research findings to students from other regions, different age groups, or other academic years.

Secondly, this survey is cross-sectional, which limits the ability to establish causal relationships. It has identified sleep conditions as risk factors for NSSI. Therefore, universities should enhanced sleep education, conduct mental health screenings, and provide psychological counseling. Finally, this study investigated aspects such as sleep conditions by using questionnaire scales; however, objective sleep measurement instruments were not employed. This lack of objective measurement has introduced biases and subjectivity into the recalled information of the survey results. Therefore, this may render the relationship between sleep conditions and NSSI inaccurate. Although the survey was anonymous, concerns remain regarding personal exposure to NSSI, as students may hesitate to disclose their true situations for various reasons.

## Data Availability

The raw data supporting the conclusions of this article will be made available by the authors, without undue reservation.
